# Muscle‐specific sirtuin1 gain‐of‐function ameliorates skeletal muscle atrophy in a pre‐clinical mouse model of cerebral ischemic stroke

**DOI:** 10.1096/fba.2020-00017

**Published:** 2020-07-03

**Authors:** Kiril Tuntevski, Ameena Hajira, Austin Nichols, Stephen E. Alway, Junaith S. Mohamed

**Affiliations:** ^1^ Department of Human Performance West Virginia University School of Medicine Morgantown WV USA; ^2^ Laboratory of Muscle Biology and Sarcopenia Department of Physical Therapy College of Health Professions University of Tennessee Health Science Center Memphis TN USA; ^3^ Laboratory of Muscle and Nerve Department of Diagnostic and Health Sciences College of Health Professions University of Tennessee Health Science Center Memphis TN USA; ^4^ Center for Muscle, Metabolism and Neuropathology Division of Rehabilitation Sciences College of Health Professions University of Tennessee Health Science Center Memphis TN USA

**Keywords:** cerebral ischemic stroke, muscle atrophy, PARP‐1, SirT1, ZNF216

## Abstract

Stroke causes severe long‐term disability in patients due to the induction of skeletal muscle atrophy and weakness, but the molecular mechanisms remain elusive. Using a preclinical mouse model of cerebral ischemic stroke, we show that stroke robustly induced atrophy and significantly decreased SirT1 gene expression in the PTA (paralytic tibialis anterior) muscle. Muscle‐specific SirT1 gain‐of‐function mice are resistant to stroke‐induced muscle atrophy and this protective effect requires its deacetylase activity. Although SirT1 counteracts the stroke‐induced up‐regulation of atrogin1, MuRF1 and ZNF216 genes, we found a mechanism that regulates the ZNF216 gene transcription in post‐stroke muscle. Stroke increased the expression of the ZNF216 gene in PTA muscle by activating PARP‐1, which binds on the ZNF216 promoter. The SirT1 gain‐of‐function or SirT1 activator, resveratrol, reversed the PARP‐1‐mediated up‐regulation of ZNF216 expression at the promoter level, suggesting a contradicted role for SirT1 and PARP‐1 in the regulation of ZNF216 gene. Overall, our study for the first‐time demonstrated that (a) stroke causes muscle atrophy, in part, through the SirT1/PARP‐1/ZNF216 signaling mechanism; (b) SirT1 can block muscle atrophy in response to different types of atrophic signals via different signaling mechanisms; and (c) SirT1 is a critical regulator of post‐stroke muscle mass.

**Abbreviations**
CCA: Common Carotid Artery
ECA: External Carotid Artery
HRP: Horseradish peroxidase
ICA: Internal Carotid Artery
MCAO: Middle Cerebral Artery Occlusion
MuRF‐1: Muscle RING finger 1
PARP‐1: Poly ADP ribose polymerase
PGC‐1α: Peroxisome proliferator‐activated receptor‐gamma coactivator‐1alpha
SirT1: Sirtuin 1
TTC: 2,3,5‐Triphenyltetrazolium chloride
UPS: ubiquitin proteasomal system
ZNF216 (ZFAND5): Zinc finger AN1‐type containing 5

## 1 INTRODUCTION

Chronic stroke is the second leading cause of death and the third cause of disability worldwide. The overall occurrence of stroke is rising exponentially regardless of age group.^1^, ^2^ Several studies have shown that stroke causes severe muscle wasting and weakness both in humans^3^, ^4^, ^5^ and animal models.^6^, ^7^ While rehabilitative therapy is the only available treatment to improve muscle strength,^8^, ^9^ higher muscle fatigability and lower muscle strength provide poor rehabilitation outcomes in stroke patients.^10^ As a result, about two‐thirds of the patients persist in a state of insufficient recovery after stroke.^11^ Studies using animal models of cerebral ischemic stroke have shown that activation of catabolic pathways, especially the ubiquitin proteasomal system (UPS), autophagy, and apoptosis in post‐stroke muscle is largely accountable for the initiation of muscle atrophy.^6^, ^7^ However, the molecular mechanisms that activate the above pathways in post‐stroke muscle are yet to be determined. Understanding such mechanisms has enormous potential for the development of novel therapeutics aimed to prevent or at least minimize the loss of muscle mass and associated weakness in stroke patients.

The ubiquitin proteasome system (UPS) is a highly regulated major protein degradation and turnover pathway in eukaryotic cells, which plays a central role in the breakdown of skeletal muscle proteins during various atrophic conditions.^12^, ^13^ This pathway is sequentially regulated by multistep reactions, which is controlled by a group of proteins/enzymes. For example, the E1, E2, and E3 enzymes are involved in the ubiquitin conjugation of target proteins, ubiquitin‐binding proteins, and 19S subunits of the proteasome that recognize the ubiquitinylated proteins, which are finally proteolyzed in the proteasome. Several studies have shown the increased expression of mRNAs encoding the components of the UPS pathway in skeletal muscle during diverse atrophic conditions.^12^, ^14^, ^15^, ^16^, ^17^ The genes that are most often up‐regulated in skeletal muscle in response to various atrophic signals include the E3 ubiquitin ligases MAFbx/Atrogin‐1 and MuRF‐1 (muscle RING finger 1), which are considered as the markers of muscle atrophy.^18^, ^19^ A recent study has shown the increased expression of these atrophic markers in mouse skeletal muscle after cerebral ischemic stroke.^7^ While various components of the UPS such as E3 ligases have been well‐studied, the mechanism of delivering ubiquitinylated proteins to the proteasome during muscle atrophy, especially in response to stroke is understudied.

SirT1, the mammalian orthologue of the yeast NAD^+^‐dependent protein deacetylase Sir2 (silent information regulator 2), is expressed in various mammalian tissues, including skeletal muscle, and serves as a sensor and regulator of the energetic status of the cell, counteracting metabolic and age‐related disorders.^20^, ^21^, ^22^ SirT1 overexpression in skeletal muscles significantly attenuates fasting and denervation‐induced muscle atrophy.^23^ Interestingly, muscle‐specific SirT1 overexpressing mice ameliorates muscle atrophy in a mouse model of Duchenne muscular dystrophy.^24^ However, the role of SirT1 in the regulation of post‐stroke muscle atrophy remains unknown. It is also unknown whether SirT1 can block muscle atrophy in response to different types of atrophic signals via different signaling mechanisms. The objective of this study is to determine the molecular mechanisms by which stroke induces muscle atrophy and the role of SirT1 in this process. We hypothesize that stroke causes muscle atrophy, in part, by inhibiting SirT1 function and SirT1 rescue preserves post‐stroke muscle mass by blocking the expression of key players of UPS.

## 2 MATERIALS AND METHODS

### 2.1 Animals

The Institutional Animal Care and Use Committee from the West Virginia University School of Medicine approved all experimental procedures. We obtained male muscle‐specific SirT1 transgenic (SirT1^+/+^) mice from Dr Guarente (MIT) and bred with age‐matched C57BL/6J female mice (The Jackson Laboratory) to generate SirT1^+/+^ and their age‐matched WT cage control mice. All mice were kept in a temperature‐controlled room on a 12‐hour light/dark cycle, with 60% humidity, and food and water ad libitum. The mice were ~20‐weeks old at the time of the experiments and 4‐5 mice were used for each experiment.

### 2.2 Transient focal cerebral ischemia and reperfusion injury

To induce transient cerebral ischemia, we performed a middle cerebral artery occlusion (MCAO) in ~25‐28 g body weight male mice. Briefly, mice were anesthetized with 1%‐1.5% isoflurane and maintained at 37°C ± 1.0 body temperature using a heating pad during the entire procedure period. A small longitudinal incision along the midline of the neck followed by another incision on the anterior cervical fascia was made to expose the sternocleidomastoid muscle on the right side. The anterior cervical muscle group and sternocleidomastoid muscle were separated to dissect and separate the right common carotid artery (CCA), the external carotid artery (ECA), and the internal carotid artery (ICA). After a nick was made in the distal region of the ECA, we inserted a standardized polyamide resin glue‐coated 6.0 nylon monofilament (Doccol Corp.) into the ECA lumen, and then advanced ~9‐9.5 mm in the ICA lumen to block MCA blood flow. After 60 minutes of occlusion, the suture was removed to achieve reperfusion. The ECA was cauterized after the nick to avoid the blood leak. After closing the incision, the mice were initially returned to the pre‐warmed cage situated on a heating pad maintained at 37°C until they recovered before they were returned to their cages and given free access to food and water. Sham mice underwent the same surgical procedure of the MCAO mice except for the insertion of filament. The vital signs for mice such as heart rate, body temperature, and breathing rate were monitored through the procedure. Mice were euthanized after 3 days of reperfusion.

### 2.3 Neurological score system

We used the modified Bederson scoring system to quantify the neurological behaviors^25^ of the mice after the MCAO surgery. The test was performed for 3 days following reperfusion. The scoring sytem was: 0 = no deficit; 1 = forelimb flexion; 2, forelimb flexion plus decreased resistance to lateral push; 3 = unidirectional circling; 4 = longitudinal spinning or seizure activity; 5 = no movement. The average score (3 days) in each group reflected the severity of neurological deficit and a higher score meant a more severe neurological deficit. We used forelimb assessments because in our hands scoring provides less variability than attempting to score the hindlimbs during a forward motion of the animals. However, as this is a bilateral deficit, we assume that neurological impairment is similar in forelimb and hindlimb musculuature as a result of the MCAO intervention.

### 2.4 TTC staining and measurement of infarct volume

Brains were harvested on day 3 and 1‐mm thick coronal slices were sectioned in a mouse brain matrix (Roboz Surgical Store). After staining with 2% 2,3,5‐triphenyltetrazolium chloride (TTC; Sigma‐Aldrich) in PBS at 37°C for 10 minutes, the pale infarctions were readily discernable from the brick‐red non‐ischemic areas and planimetric measurements were obtained using the ImageJ software (National Institutes of Health). The calculated lesion volume was corrected for brain swelling as described by Ginsberg et al.^26^

### 2.5 Mean fiber cross‐sectional area measurements

Muscle fiber cross‐sectional area (CSA) analysis was performed in the tibialis anterior (TA) muscles of sham and stroke mice. The TA muscles were collected, mounted to cork blocks using tissue freezing medium (OCT, Thermo Fisher Scientific), flash frozen using isopentane cooled in liquid nitrogen, and then stored at −80°C. Frozen tissue cross sections measuring 10 μm thick were cut from the mid‐belly of the muscles using a cryostat (Leica CM1950, Leica CM1950), mounted on charged microscope slides (Fisher Scientific), and stored at −80°C. For staining, the slides were air‐dried 10 minutes, hydrated in 1× PBS for 5 minutes, and incubated for 60 minutes in 10% normal goat serum (Vector laboratories) at RT. The tissue sections were then incubated overnight at 4°C with the rabbit anti‐Laminin primary antibody (Sigma). After washing three times in 1× PBS, the sections were incubated in anti‐rabbit Alexa Fluor 555 secondary antibody (Thermo Fisher Scientific) for 1 hour at RT. After washing in 1× PBS for three times (5 minutes each), the sections were mounted with Prolong Gold antifade mounting media (Thermo Fisher Scientific) for imaging with a Zeiss LSM 510 Meta confocal microscope confocal microscope at WVU imaging facility. Muscle fiber CSAs were traced and measured manually (∼300 fibers per muscle) in ImageJ v.1.43.

### 2.6 Western blot

The TA muscle was removed and frozen in liquid nitrogen and then stored at −80°C. Western blots were performed from the mid‐belly region of the TA as described previously.^22^, ^27^, ^28^ Briefly, muscle tissues or cell pellets were lysed in RIPA buffer (Pierce) containing protease and phosphatase inhibitors using a glass Teflon homogenizer and the supernatant was collected by centrifugation at 12 000 rpm for 10 minutes. After protein quantification by Barford assay, 20 μg proteins were resolved in 4%‐12% gradient NuPAGE Bis‐Tris SDS‐PAGE gels (ThermoFisher Scientific) at 120 V for 30 minutes in NuPAGE MES SDS running buffer (ThermoFisher Scientific). Proteins were transferred to a nitrocellulose membrane for 15 minutes at 15 V with a Trans‐Blot SD semi‐dry transfer cell (Bio‐Rad). After blocking with 2% Amersham ECL Blocking Agent (Global Life Sciences Solutions) in TBST, membranes were incubated with primary antibodies (1:000 dilution) overnight at 4°C. After washing in TBST, the membranes were incubated in HRP linked anti‐mouse or anti‐rabbit secondary antibody (1:5000) for 1 hour at RT. HRP signals were developed with an ECL substrate (ThermoFisher Scientific) and imaged using a G:Box Bioimaging System (Syngene). The primary and secondary antibodies information: SirT1 (Cat# 09‐843) and PGC‐1α (Cat# ST1202) were purchased from Millipore; PARP‐1 (Cat# ab32138) and RNA Polymerase II (Cat# ab240740) were purchased from Abcam. GAPDH (Cat# 5174), Acetylated lysine (Cat# 9441), ploy (ADP)R (Cat# 83732), anti‐rabbit IgG‐HRP (7074S), and anti‐mouse IgG‐HRP (7076S) were purchase from Cell Signaling.

### 2.7 Quantitative PCR (qPCR)

qPCR was performed as described previously.^27^, ^28^ Briefly, total RNA was extracted from tissues or cell pellets using Trizol Reagent (Thermo Fisher Scientific). Total RNA (1 μg) was reverse‐transcribed using SuperScript III First‐Strand Synthesis SuperMix according to the manufacturer's protocols (Thermo Fisher Scientific). PCRs were performed using SensiMix SYBR Hi‐ROX Kit according to the manufacturer's instructions (Bioline). qPCRs were performed on an AB7300 qPCR system (Thermo Fisher Scientific). The temperature cycle profile for the qPCR reactions was 95°C for 15 minutes and 40 cycles of 94°C for 15 seconds, 60°C for 30 seconds, and 72°C for 30 seconds. Melting curve analysis was also included at one cycle of 95°C for 1 minutes, 55°C for 30 seconds, and 95°C for 30 seconds to verify the specificity of the amplified PCR products. The number of amplified transcripts (2^ΔΔ^ CT) was estimated by the comparative CT (^Δ^CT) method and normalized to an endogenous reference (GAPDH) relative to a calibrator. All PCR products were verified on an agarose gel stained with ethidium bromide to discriminate between the correct amplification products and the potential primer dimers. Primer sequencing information: GAPDH forward 5′‐GAAGGTGAAGGTCGGAGTCA‐3′ and reverse 5′‐TGGAAGATGGTGATGGGATT‐3′; SirT1 forward 5′‐TGCTGGCCTAATAGAGTGGCA‐3′ and reversed 5′‐CTCAGCGCCATGGAAAATGT‐3′; Atrogin‐1 forward 5′‐AACCGGGAGGCCAGCTAAAGAACA‐3′ and reverse 5′‐TGGGCCTACAGAACAGACAGTGC‐3′; MuRF‐1 forward 5′‐GAGAACCTGGAGAAGCAGCT‐3′ and reverse 5′‐CCGCGGTTGGTCCAGTAG‐3′; ZNF216 forward 5′‐CCCATGCTGTGTAGTACAGGA‐3′ and reverse 5′‐ GCTCATTCTGCCACTATTCTGC‐3′; PARP‐1 forward 5′‐GGCAGCCTGATGTTGAGGT‐3′ and reverse 5′‐GCGTACTCCGCTAAAAAGTCAC‐3′.

### 2.8 Primary myoblast isolation, culture, and differentiation

For in vitro studies, we used primary myoblasts, isolated from young mouse hind limb muscles, and cultured them to induce myotube formation as we described previously.^22^, ^29^ Briefly, hindlimb muscles, from 4‐week old C57BL/6J mice, were excised and cut into small pieces followed by incubation in 1500 U collagenase type II (Worthington Biochemical) in Dulbecco's modified Eagle's medium (DMEM) containing 10% horse serum. After removing the digestion medium, the partially digested muscle pieces were incubated in 300 U collagenase type II with 6 U dispase (Thermo Fisher Scientific) in DMEM containing 10% horse serum. After trituration, the resulting supernatants were collected by brief centrifugation, filtered through 40 μm cell strainers and then washed in 1× PBS containing 0.025% sodium azide. The resulting mononuclear cells were blocked in a 10% normal goat serum (Vector Labs). To separate the satellite cells from the mononuclear cells, we used fluorophore‐conjugated primary antibodies specific for the following cell surface markers: CD31 (‐APC), Sca‐1 (‐PacBlue), CD45 (‐PE‐Cy7), and VCAM‐1 (‐Biotin) (BioLegend). A second incubation was used to bind Rhodamine‐conjugated avidin (Vector Labs) to the biotin‐conjugated antibody. After washing, the fluorophore‐conjugated cells were resuspended in fetal bovine serum and VCAM^+^/CD31^−^/ CD45^−^/Sca‐1^−^ satellite cells were isolated by a FACSCalibur flow cytometer equipped with a 15‐molecular weight (MW) 488 nm argon laser and 633 nm red diode laser (Becton and Dickinson). After cell counting, 4000‐10 000 cells were cultured in a 60‐mm collagen‐coated culture dish containing growth medium (GM; 80% Ham's F‐12, 20% fetal calf serum, 0.025% basic fibroblast growth factor in 0.5% BSA, 100 units/mL penicillin, and 100 μg/mL streptomycin) at 37°C and 5% CO_2_. At 70%‐80% confluence, the proliferating cells were plated in a new dish and cultured in fresh GM. To induce differentiation into myotubes, the GM was replaced with DM (differentiation media) containing 10% horse serum and antibiotics.

### 2.9 siRNA transfection

Myotubes were transfected with PARP‐1 siRNA as we published before.^21^ Briefly, myotubes were grown in Opti‐MEM I medium (Invitrogen) for 24 hours before transfection with 500 pmol of siRNA specific for mouse PARP‐1 or nonspecific siRNA (Santa Cruz Biotechnology). siRNA transfection was performed with Lipofectamine RNAi MAX as per the manufacturer's guidelines (Thermo Fisher Scientific). The transfection medium was replaced with DM after 8 hours. Subsequent assays were conducted 24‐48 hours after transfection.

### 2.10 Chromatin Immunoprecipitation (ChIP) assays

ChIP assays were performed as described previously.^28^ Briefly, muscle tissues or cell pellets were cross‐linked in 37% formaldehyde (10 μL/mL) for 15 minutes at RT followed by the addition of glycine to a final concentration of 125 mmol/L. Samples were then washed twice in PBS and pelletized by centrifugation at 700 *g* at 4°C for 5 minutes in PBS containing protease inhibitor. The pellets were lysed in 1 mL of lysis buffer (1% SDS, 10 mmol/L EDTA, and 50 mmol/L Tris, pH 8.1) and sonicated on ice to shear DNA to an average length of 200‐ to 1000‐bp fragments. The sheared DNAs were precleared by centrifugation at 10 000 *g* at 4°C for 10 minutes and split into 100‐μL aliquots. To each of the 100‐μL aliquots, 900 μL dilution buffer (0.01% SDS; 1.1% Triton X‐100; 1.2 mmol/L EDTA; 16.7 mmol/L Tris‐HCl, pH 8.1; and 167 mmol/L NaCl) and 60 μL protein G agarose were added and incubated at 4°C for 1 hour with rotation. The Protein G agarose was pelleted by centrifugation at 3000 *g* 4°C for 1 minute, and the supernatant was collected, followed by incubation with either 5 μg of affinity‐purified anti‐PARP‐1, anti‐RNA Polymerase II, or normal IgG (negative control) at 4°C for overnight with rotation (before incubation, 10 μL supernatant was removed as input and stored at 4°C). The protein‐DNA complexes were then collected by the addition of 60 μL protein G agarose and incubated at 4°C for 1 hour, followed by 1‐minute centrifugation at 5000 *g*. The pellets were washed in 1 mL each of the following cold wash buffers in the following order: low‐salt buffer (1% SDS; 1% Triton X‐100; 2 mmol/L EDTA; 20 mmol/L Tris‐HCl, pH 8.1; and 150 mmol/L NaCl), high‐salt buffer (1% SDS; 1% Triton X‐100; 2 mmol/L EDTA; 20 mmol/L Tris‐HCl, pH 8.1; and 500 mmol/L NaCl), LiCl buffer [0.25 mol/L LiCl, 1% IGEPAL‐CA630, 1% deoxycholic acid (sodium salt), 1 mmol/L EDTA, and 10 mmol/L Tris‐HCl, pH 8.1], and TE buffer (10 mmol/L Tris‐HCl and 1 mmol/L EDTA, pH 8.0). During every wash, the suspension was incubated for 5 minutes with rotation and centrifuged at 5000 *g* for 1 minutes, and the supernatant was discarded. The protein‐DNA complex was eluted by resuspending the pellet with 200 μL elution buffer (10 μL 20% SDS, 20 μL 1 mol/L NaHCO_3_, and 170 μL sterile distilled water). Crosslinks of the protein‐DNA complexes were reversed by the addition of 8 μL 5 mmol/L NaCl, incubating at 65°C for 5 hours, and addition of 1 μL RNase A followed by incubation at 37°C for 30 minutes. The DNA was analyzed by qPCR using ZNF216 or GAPDH promoter‐specific primers. PCR amplification was also performed from an input sample that represents 1% of the total input chromatin.

### 2.11 Statistical analysis

The results are expressed as means ± SEM. Comparisons among different groups were performed by appropriate ANOVA (1‐way or 2‐way) followed by a Bonferroni post hoc test if a main effect was identified. Paired data were evaluated by Student t test. A *P* value of ≤0.05 was considered statistically significant.

## 3 RESULTS

### 3.1 Stroke reduces SirT1 functions in skeletal muscle

**FIGURE 1:**
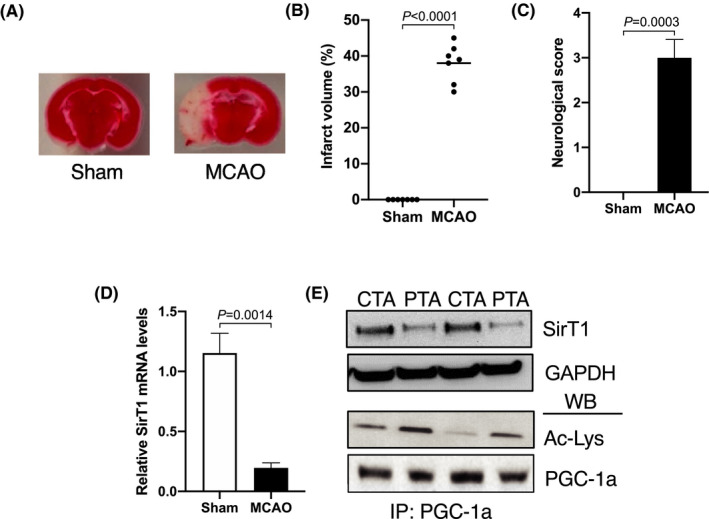
Stroke causes severe muscle atrophy and represses SirT1 gene expression. Male C57BL/6J mice, ~20‐w old, were subjected to 60’MCAO or sham (same procedures of 60’MCAO mice except for the placement of occlusive filament that will cause MCAO) surgery followed by 3 days of reperfusion. A, Representative TTC stained 1 mm coronal brain sections of mid cortex region after 60’MCAO followed by 3‐d reperfusion or sham surgery indicating areas of healthy (red) and ischemic injury (white marked area) tissues. B, Size of the lesion in the ipsilateral hemisphere expressed as a % of the total contralateral hemisphere volume (n = 5). C, Neurologic deficits were measured for 3 days by the mNSS (0 is normal; n = 5/group). On day 3 after surgery, the paretic tibialis anterior (PTA) or the corresponding TA (CTA) muscle of sham mic were collected for total RNA or cell lysate extraction. D, qPCR data showed mRNA expression of the SirT1 gene (n = 4). E, Representative immunoprecipitation and western blot images showed SirT1 protein expression and its activity (n = 4). Values in each graph indicate the mean ± SEM [Color figure can be viewed at wileyonlinelibrary.com]

To determine the regulation of the SirT1 gene in skeletal muscle in response to stroke, we measured SirT1 mRNA and protein levels in the paralytic tibialis anterior (PTA) muscle of MCAO mice and corrsponding tibialis anterior (CTA) muscle of sham mice. 60’MCAO followed by the 3 days reperfusion significantly induced brain lesion (~45%) in the lateral striatum and parietal cortex regions (Figure 1A,B). The induction of the brain lesion severely affected motor function as evidenced by an impaired neurological score (Figure 1C). Moreover, 60’MCAO significantly decreased both SirT1 mRNA (Figure 1D) and protein (Figure 1E) expressions in PTA muscle compared to CTA muscle of sham mice. PGC‐1α (peroxisome proliferator‐activated receptor‐gamma coactivator‐1α) is the known and well‐established target protein of SirT1.^30^ The decreased SirT1 level in PTA muscle as compared to CTA muscle was accompanied by the decreased SirT1 activity as evidenced by increased PGC‐1α acetylation levels (Figure 1E). These data suggest that stroke decreases SirT1 gene expression and its activity and induces atrophy in skeletal muscle.

### 3.2 SirT1 gain‐of‐function prevents stroke‐induced muscle atrophy

**FIGURE 2:**
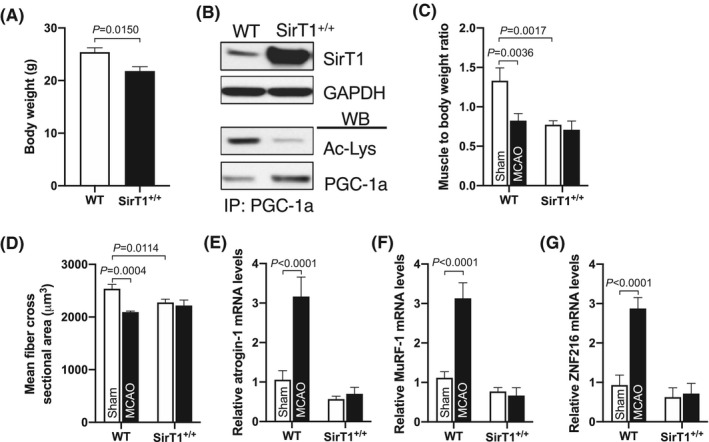
SirT1^+/+^ mice are resistant to post‐stroke muscle atrophy. Male, ~20‐w old, SirT1^+/+^ and their age‐matched WT (C57BL/6J) control mice were subjected to 60’MCAO or sham surgery followed by 3 d of reperfusion. RNA or cell lysate were extracted from paretic tibialis anterior (PTA) or the corresponding TA (CTA) muscle of sham mice of WT and SirT1^+/+^ mice. A, Mean body weight of WT and SirT1^+/+^ mice (n = 6). B, SirT1 protein expression and its activity in the TA muscles of WT and SirT1^+/+^ mice (n = 4). C‐D, Mean CTA and PTA muscle mass and fiber cross‐sectional areas of WT and SirT1^+/+^ mice (n = 4). E‐G, qPCR data showed atrogin‐1 (E), MurF‐1 (F), and ZNF216 (G) mRNA levels in PTA and CTA muscle of WT and SirT1^+/+^ mice (n = 4). Values in each graph indicate the mean ± SEM

As stroke inhibits the expression of SirT1, which has been shown to block muscle atrophy,^23^, ^24^ we rationalized that preserving SirT1 function may prevent post‐stroke muscle atrophy. To test this, we conducted MACO in a muscle‐specific SirT1 gain‐of‐function (SirT1^+/+^) mouse model. We did not observe any significant abnormal gross phenotype appearance between WT and SirT1^+/+^ mice. The whole‐body weight of SirT1^+/+^ mice was markedly less compared to WT mice (Figure 2A). As expected, SirT1 protein levels were higher in the skeletal muscle of SirT1^+/+^ mice compared to WT mice (Figure 2B). The increased SirT1 levels were followed by the decreased PGC‐1α acetylation levels in skeletal muscle (Figure 2B). These data indicate that both SirT1 level and activity were high in SirT1^+/+^ mice compared to WT mice. This observation strongly supports the rationale for using this trangenic mouse model in the current study to evaluate a role for SirT1 in the regulation of post‐stroke muscle mass. PTA muscle from SirT1^+/+^ mice displayed no loss of muscle mass (Figure 2C). Mean TA muscle fiber's cross‐sectional area (FCA) was greater in SirT1^+/+^ mice than FCA in the CTA muscle of WT mice (Figure 2D). However, the basal muscle mass and FCA was less in SirT1^+/+^ mice compared to WT mice, which had a sham surgery. PTA muscle from WT mice showed the elevated expression of Atrogin‐1 (Figure 2E) and MuRF‐1 genes (Figure 2F), but these genese were not elevated in the PTA muscle of SirT1^+/+^ mice (Figure 2E,F). Moreover, PTA muscle from WT mice displayed higher expression of the ZNF216 gene, a protein that identifies and dispenses the ubiquitylated proteins to the proteasome for degradation,^31^ compared to SirT1^+/+^ mice where ZNF216 levels were lower in PTA muscle (Figure 2G). These data indicate that stroke activates the key players of UPS and causes severe skeletal muscle atrophy in WT mice. In contrast, stroke has a minimal effect on signaling for muscle atrophy in SirT1^+/+^ mice, suggesting that SirT1 offsets the stroke‐induced muscle atrophy program.

### 3.3 PARP‐1 regulates ZNF216 gene

**FIGURE 3:**
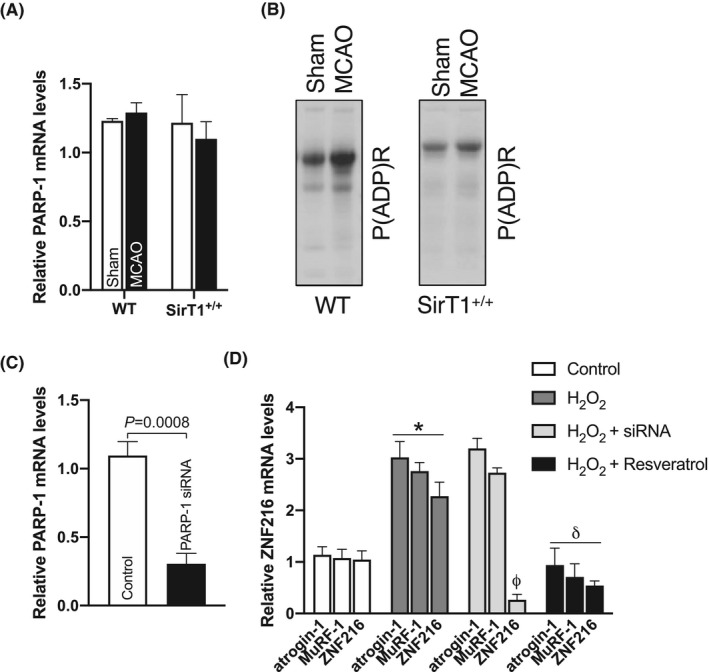
PARP‐1 regulates the ZNF216 gene. Male, ~20‐ w old, SirT1^+/+^ mice and their age‐matched WT (C57BL/6J) control mice were subjected to 60’MCAO or sham surgery followed by 3 d of reperfusion. RNA or cell lysate were extracted from paretic tibialis anterior (PTA) or the corresponding TA (CTA) muscle of sham mice in WT and SirT1^+/+^ mice (n = 4). A, qPCR data showed PARP‐1 mRNA levels in the PTA and CTA muscles of WT and SirT1^+/+^ mice (n = 4). B, Representative western blot images showed global protein parylation (PARP activity) in the TA muscles of WT and SirT1^+/+^ mice (n = 3). C, Myotubes generatred from primary myoblasts were transfected with PARP‐1 siRNAs or control siRNAs for 48 h and total RNA was isolated and used in qPCR data to measure PARP‐1 mRNA levels. D, PARP‐1 siRNA transfected myotubes were treated with 125 μmol/L of H_2_O_2_ with or without resveratrol for 4 h to measure ZNF216 mRNA levels in the total RNA using qPCR (n = 4). Values in each graph indicate the mean ± SEM. * indicates comparison vs control. φ and δ indicate the comparison of the specific gene in the H_2_O_2_‐treated group

Our data along with the work of others have shown that SirT1 and PARP‐1 compete with each other for the intracellular NAD^+^ pool and the higher activity of one enzyme inhibits the activity of other.^20^, ^21^, ^32^, ^33^ In the current study, stroke represses SirT1 activity and that loss of SirT1 function may increase the activity of PARP‐1 in post‐stroke muscle. Moreover, PARP‐1 has been shown to induce muscle atrophy in response to various atrophic cues.^34^ Thus, it is reasonable to determine the regulation and role of PARP‐1 in post‐stroke muscle. Surprisingly, 60’MCAO did not alter PARP‐1 gene expression in PTA muscle of both WT and SirT1^+/+^ mice (Figure 3A). Contrarily, stroke considerably elevated global PARP activity in the PTA muscle of WT mice, but not in the PTA muscle of SirT1^+/+^ mice, which inhibited PARP activity, as evidenced by reduced levels of global protein parylation (Figure 3B). These data suggest that stroke increases PARP activity without changing PARP‐1 gene transcription and SirT1 inhibits stroke‐induced PARP activity. We and others have shown that H_2_O_2_ is an established stimulator of PARP‐1 activity.^21^, ^35^ Thus, we used H_2_O_2_ to activate PARP‐1 and PARP‐1 siRNAs to knockdown PARP‐1 protein levels to determine its role in the regulation of the key players of UPS. Primary myotubes from primary myoblasts transfected with PARP‐1 siRNAs for 48h robustly inhibited PARP‐1 mRNA levels (Figure 3C), suggesting the specificity of siRNAs. We incubated myotubes with H_2_O_2_ (125 μmol/L for 6 hours) and measured atrogin‐1, MuRF‐1, and ZNF216 gene expressions, which were significantly elevated compared to control (Figure 3D). Transfection of primary myotubes with the PARP‐1 siRNA before H_2_O_2_ treatment substantially decreased ZNF216 mRNA levels, but not atrogin‐1 and MuRF‐1 levels (Figure 3D). These data indicate that PARP‐1 is upstream of ZNF216 and selectively regulates the components of UPS. Finally, we determined if activation of SirT‐1 will reverse the ZNF216 gene expression. Treatment of myotubes with the SirT‐1 activator resveratrol (25 μmol/L for 24 hours) significantly reduced H_2_O_2_‐mediated atrogin‐1, MuRF‐1 and ZNF216 mRNA expressions (Figure 3D). These results indicate that SirT‐1 and PARP‐1 have a contradictory role in the regulation of the ZNF216 gene expression, but not atrogin‐1 and MuRF‐1 regulations, which are controlled by SirT1.

### 3.4 PARP‐1 binds on the ZNF216 promoter

**FIGURE 4:**
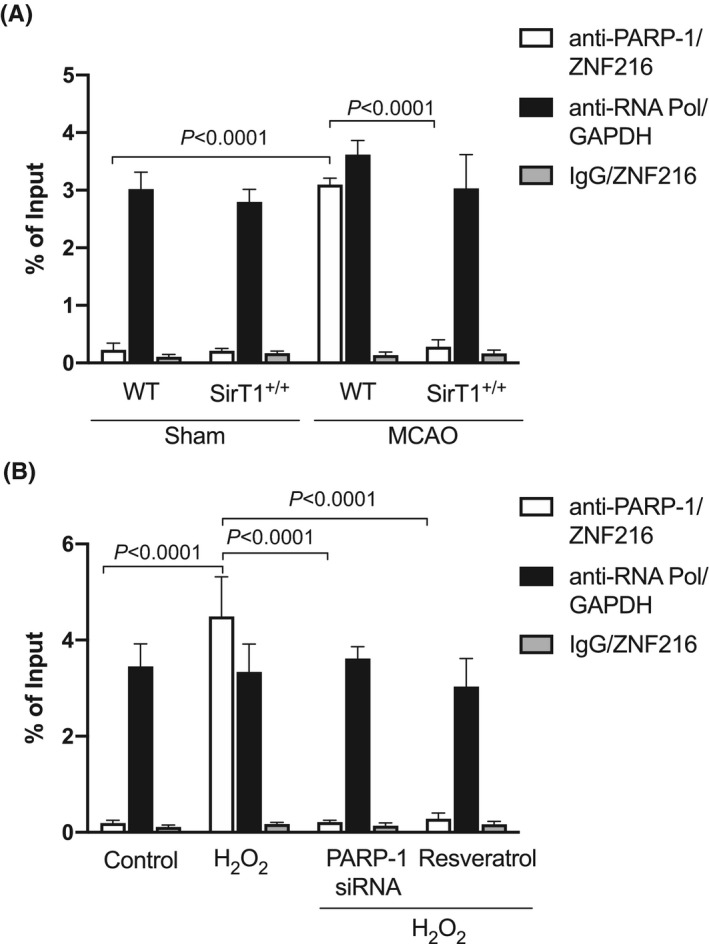
PARP‐1 binds on ZNF216 promoter. Male, ~20‐w old, SirT1^+/+^ and their age‐matched WT (C57BL/6J) control mice were subjected to 60’MCAO or sham surgery followed by 3 d of reperfusion. B‐C, Chromatin was isolated from paretic tibialis anterior (PTA) or the corresponding TA (CTA) muscle of sham mice from WT and SirT1^+/+^ mice (B) or PARP‐1 siRNA transfected myotubes treated with 125 μmol/L of H_2_O_2_ with or without resveratrol (C) and precipitated with anti‐PARP‐1, anti‐RNA Poly II, or nonspecific IgG. qPCRs were performed with primers specific for ZNF216 or GAPDH. Values in each graph indicate the mean ± SEM (n = 3)

To determine the mechanism by which PARP‐1 regulates the ZNF216 gene, we measured the activity of PARP‐1 on the ZNF216 promoter because PARP‐1 has been shown to regulate gene transcriptions in addition to their protein PARylation function.^36^, ^37^ Our ChIP assay followed by qPCR showed a dramatic increase in the binding activity of PARP‐1 on the ZNF216 promoter in PTA muscle of WT mice in contrast to the PTA muscle of SirT1^+/+^ mice, which showed decreased levels of PARP‐1 binding activity on the ZNF216 promoter (Figure 4A). These data suggest that stroke up‐regulates ZNF216 gene expression through PARP‐1 that binds on the ZNF216 promoter. Next, we determined the effect of PARP‐1 inhibition on the ZNF216 promoter. Myotubes transfected with H_2_O_2_ showed higher PARP‐1 binding activity on the ZNF216 promoter compared to control, whereas transfection of myotubes with PARP‐1 siRNAs or resveratrol revoked the H_2_O_2_‐induced PARP‐1 binding activity on the ZNF216 promoter (Figure 4B). These data suggest a role for PARP‐1 in the regulation of the ZNF216 promoter activity.

## 4 DISCUSSION

In the present study, we have identified the molecular mechanism that causes muscle atrophy in cerebral ischemic stroke. Especially, we focused our study to determine how the stroke activates the key players of UPS. We found that cerebral ischemic stroke by 60’ MCAO induced severe muscle atrophy and significantly increased the expressions of the markers of UPS such as MuRF1, Atrogin1, and ZNF216 genes in skeletal muscle. We also found that stroke inhibits SirT1 gene expression and its activity in skeletal muscle. Interestingly, SirT1 rescue in post‐stroke muscle, dramatically reduced atrophy and the expression of MuRF1, Atrogin1 and ZNF216 genes. We further demonstrate that stroke activates PARP‐1 protein that increases muscle levels of ZNF216, but not MuRF1 and Atrogin1 gene expressions at the transcriptional level. Finally, we confirmed that SirT1 inhibits ZNF216 gene expression by blocking PARP‐1 activity and that inhibition likely plays a role in reversing post‐stroke muscle atrophy. These findings provide experimental evidence for the first time that SirT1 deacetylase activity is essential for preserving post‐stroke muscle mass and perhaps associated function.

Muscle atrophy as a result of elevated protein breakdown in skeletal muscle has been observed in many disease conditions, including stroke.^6^, ^7^ In muscle atrophy, activation of the UPS plays a crucial role in protein breakdown.^12^, ^13^ Several studies have shown the increased expressions of mRNAs encoding UPS genes in atrophying muscle,^12^, ^14^, ^15^, ^16^, ^17^ in particular, the E3 ubiquitin ligases Atrogin‐1 and MuRF‐1.^18^, ^19^ In the current study, we have shown the elevated levels of these E3 ubiquitin ligases in post‐stroke muscle, which is consistent with the previous observation.^7^ However, the molecular mechanisms that regulate these atrophy markers in post‐stroke muscle remain unknown. Interestingly, we found a role for SirT1 in the regulation of stroke‐induced Atrogin‐1 and MuRF‐1 in post‐stroke muscle. Precisely, muscle‐specific SirT1 gain‐of‐function mice are resistant to the stroke‐induced muscle atrophy as evidenced by no changes in the muscle mass and the expression levels of Atrogin‐1 and MuRF‐1 genes. An earlier study has shown that SirT1 overexpression in skeletal muscles significantly attenuates fasting and denervation‐induced muscle atrophy by blocking the activation of the FoxO transcription factors, which regulate MuRF1 and Atrogin‐1 gene expressions.^23^ However, we could not find the binding activities of the FoxO transcription factors on the MuRF1 and Atrogin‐1 promoters in the post‐stroke muscle (data are not shown). This suggests that SirT1 can block muscle atrophy in response to different types of atrophic signals via different signaling mechanisms. Furthermore, the mechanism of how ubiquitinylated proteins are carried to the proteasome is largely unknown. A recent study has shown that the expression of the ZNF216 protein is elevated in atrophic skeletal muscle and ZNF216‐deficient mice show resistance to muscle atrophy, which is accompanied by abnormal accumulation of polyubiquitinylated proteins in skeletal muscle.^31^ This suggests that ZNF216 plays a role in anchoring ubiquitinylated proteins to the proteasome. However, the molecular mechanism that increases the ZNF216 gene expression in skeletal muscle during atrophy is poorly studied. We demonstrate that SirT1 overexpression decreased the stroke‐induced ZNF216 gene expression in skeletal muscle. These findings provide the essential evidence that SirT1 inhibits the ZNF216 gene transcription, in addition to Atrogin‐1 and MuRF‐1 and is critical for blocking UPS activation in skeletal muscle during atrophic conditions.

PARP‐1 is a main mediator of the response to cellular stress induced by physiological stressors. Although the basal activity of PARP‐1 is necessary to maintain genome integrity and cellular homeostasis in stress, overactivation of PARP‐1 induces skeletal muscle atrophy that is more common in disease/disorder conditions. For example, it has been shown that overactivation of PARP causes deleterious effects on tissues mainly due to the depletion of intracellular NAD^+^ and ATP stores that lead to cell dysfunction and death.^38^ Notably, this mechanism of cell destruction also occurs during muscle wasting induced by various chronic disease conditions, such as acute lung and renal injuries,^39^, ^40^, ^41^ and sepsis.^42^ In line with these observations, our current study found an over activation of PARP in skeletal muscle after stroke. On the other hand, we and others have shown that PARP‐1 or PARP‐2 inhibition promotes oxidative metabolism and increases energy expenditure in skeletal muscles^20^, ^21^, ^32^, ^33^ and attenuates muscle mass loss in cancer cachectic mice through epigenetic regulation.^43^ Although these studies have shown a role for PARP in the regulation of muscle mass, the molecular mechanisms by which PARP promotes muscle atrophy are unknown. In the current study, we found a novel mechanism for PARP‐1 in promoting muscle atrophy, especially in post‐stroke muscle. We demonstrate that stroke activates PARP‐1 that binds on the ZNF216 promoter to increase the ZNF216 gene expression. We further demonstrate that inhibition of PARP activity by siRNA strategy reversed the H_2_O_2_‐induced ZNF216 gene expression. These data provide experimental evidence that stroke causes muscle atrophy, in part, through PARP‐1‐regulated ZNF216 protein and inhibition of PARP‐1 activity in the post‐stroke muscle may have a beneficial effect in reversing the stroke‐induced muscle loss through ZNF216 inhibition. In the present study, we also showed that while stroke inhibits SirT1 activity, it increases PARP activity in skeletal muscle. In contrast, overexpression of SirT1 in skeletal muscle prevented the elevation of stroke‐induced PARP activity. Also, pre‐treatment of myotubes with resveratrol blocked the H_2_O_2_‐induced PARP activities. Collectively, these studies suggest that the loss of SirT1 function in post‐stroke muscle is likely due to overactivation of PARP‐1 that could have utilized most of the available intracellular NAD^+^ needed for SirT1 activity. Moreover, stroke inhibits SirT1 gene transcription as SirT1 mRNA and protein levels were declined in PTA muscle. This suggests that stroke negatively regulates SirT1 both at the transcriptional and post‐transcriptional levels.

In summary, we demonstrate that SirT1 plays an essential role in preventing cerebral ischemic stroke‐induced muscle atrophy. The loss of SirT1 function in post‐stroke muscle is primarily responsible for the activation of UPS at least through elevating Atrogin‐1, MuRF1, and ZNF216 gene expressions. While we found a mechanism by which stroke elevates ZNF216 gene expression, via PARP‐1 and could not find the binding activity of FoxO1 or FoxO3a on Atrogin‐1 and MuRF‐1 promoters, the mechanisms by which SirT1 prevents Atrogin‐1 and MuRF‐1 gene expression, and how stroke represses SirT1 gene transcription is yet to be discovered.

## AUTHOR CONTRIBUTION

K. Tuntevski, A. Hajira, and A. Nichols contributed to the animal and in vitro experiments. J. S. Mohamed designed the studies, supervised the project, analyzed the data, and wrote the manuscript. S. E. Alway provided overall assistance and edited the manuscript.
